# Bacteriology

**Published:** 1904-08-27

**Authors:** 


### BACTERIOLOGY.

**Leishman-Donovan Bodies.**—Rogers[1] has examined the blood drawn off during life from the spleen of 30 patients suffering from prolonged fever in the district of Dinajur. In 10 of these Leishman-Donovan bodies were found, and there was also in each a large mononuclear increase. He has also found the same parasite in the blood of cases of Kala-azar, and suggests that all these chronic fevers showing Leishman-Donovan bodies should be classed under the one title, "cachexial fevers." The commonest form of the parasite is that of a small oval body, the longest diameter being about one third of the diameter of a red blood corpuscle. There are two nuclei close together, one small and rod-shaped, and staining deeply, and the other round and short, and staining faintly. They are free in the blood. Manson and Low[2] in a short note announce that they have discovered in a case of Kala-azar the Leishman-Donovan bodies in the spleen and liver blood during life, and in the spleen, liver, bone-marrow, and mesenteric glands after death. Neave[3] records the finding of large numbers of these parasites in the blood of a boy who had come to Khartoum from the Bahr-el-Gazal. In this case the blood of the patient showed 67 per cent. of large mononuclear cells. This is the second reported case of the disease in Africa. On July 2nd a further communication appeared in the *British Medical Journal* from Manson and Low in reference to the case of Kala azar which we have mentioned above. Examination of the intestines showed ulceration of the ileum and colon and patchy areas of atrophy in the mucous membrane. Sections made at the ulcerated surfaces showed Leishman-Donovan bodies enclosed in cells, and although the organisms are never found within the blood cells they are in tissues generally intracellular. The writers think that possibly it is through an ulcerated surface that the Leishman-Donovan bodies escape from the body. Dysenteric ulceration is a very usual termination of Kala-azar. In the journal of the same date there is a telegram from Rogers which states that he has succeeded in cultivating trypanosomata from Leishman bodies. No details are as yet supplied, but it is not unlikely that his work may confirm the view that these bodies are only a resting stage of a trypanosome. Schaudiun has recently shown that the trypanosoma noctuæ has an alternately non-flagellated intracellular stage and a flagellated extra-cellular stage. Grieg and Gray[4] recommend that in cases of trypanosomiasis and sleeping sickness the juice from enlarged lymphatic glands should be sucked out by means of a hypodermic needle and then examined in the fresh state for parasites. Using this method they have found that the discovery of the parasite is much facilitated, actively moving trypanosomes being readily detected, as they are often present in the lymph tissue in large numbers. They were never able by this method to detect diplo- or streptococci. The blood of their patients showed a constant increase in the percentage of lymphocytes but the total leucocytes was not increased. Laveran[5] has recently described two varieties of trypanosome which are apparently new. One measures $25\mu$ by $2\mu$, and excites a rapidly fatal form of emaciation and fever in dromedaries, and the second produces the disease known as "souma" in cattle.

**The Rheumatic Diseases.**—Poynton[6] in a review of some recent writings upon arthritis mentioned that he and Paine have produced iritis in rabbits by injecting cultures of the diplococcus rheumaticus into the auricular vein. This result was also accomplished by Shaw, and he in one instance by repeated doses induced a relapse. Phillips, who made an exhaustive bacteriological examination of 26 cases of acute rheumatism, was unable to demonstrate micro-organisms in any of them, either by cultures or by inoculations. McCrae,[7] in a detailed report of 110 cases of osteo-arthritis, states that he has never been able to isolate micro-organisms from the tissues or joint fluids in this disease. He regards the fact that the lymph glands near affected joints are often enlarged as corroborative of an infective origin, and this is confirmed in his opinion by the early joint changes being inflammatory rather than degenerative.

**Modification of Bacilli.**—It has long been known that old cultures of typhoid bacilli lose their motility, and Stephens[8] in an interesting communication shows that this is due to loss of their flagellæ, and that by passage through a guinea-pig a new motile flagellate generation can be secured. Indeed, it seems certain that this bacillus and others may appear under a great variety of complex forms. Ainley-Walker and Murray[9] show that by growing the typhoid bacillus in a liquid or solid medium containing ·2 per cent. of saturated alcoholic solution of methyl violet or other dye the normal short bacillus is converted into a form consisting of enormously long filaments or threads. These threads which may stretch across the field of a one-twelfth oil immersion lens are not segmented, but are supplied with numerous flagellæ throughout their length, and present numerous examples of dichotomy (true branching). The organism resumes its ordinary shape when restored to common media. Similar results were obtained with other organisms such as bacillus coli and the cholera vibrio.

**Organisms in Healthy Tissues.**—Morgan,[10] in a review of the literature on this subject, shows what a great diversity of opinion exists as to the presence of bacteria in normal tissues. This has, in part, been due to the fact that insufficient time of incubation was given, Ford's work showing that where the incubation period was prolonged for from 12 to 17 days the organisms had an opportunity of growing out from the pieces of tissue and appearing on the medium. The organisms he found were staphylococcus albus and aureus, B. mesentericus, B. proteus, B. subtilis, and B. coli. Of 96 organs taken from 34 animals only 30 per cent. were sterile. Morgan has repeated these experiments using rabbits, taking the utmost care to prevent contamination, and giving prolonged periods or ærobic and anærobic incubation. In this way 80 organs from 10 rabbits were examined. Growth in ærobic broth cultures was obtained from 23 organs, the bacteria were non-pathogenic, and included B. mesentericus vulgatus and other spore-bearing forms. No anærobic forms were cultivated. Subsequent experiments showed that these organisms were probably accidental contaminations from the air at the time of the post-mortem examination, and he concludes that normal organs do not contain bacteria. Forzosek[11] added cultures of B. prodigiosus, B. fluorescens, B. violaceus, and B. pyocyaneus to the food of certain animals and found them again in from 30 to 50 per cent. of the animals killed from five to 20 days later. This result, however, is not strictly comparable with the above as the organisms introduced might cause gastro-intestinal disturbance.

